# 超高效液相色谱-串联质谱法定量测定低致敏婴幼儿配方乳粉中牛乳过敏原

**DOI:** 10.3724/SP.J.1123.2024.12030

**Published:** 2025-11-08

**Authors:** Daokun XU, Yang YANG, Haolun HUANG, Xiaojie SUN, Wenyan HU, Xinmei LIU, Jun YANG

**Affiliations:** 1.南京市食品药品监督检验院，江苏 南京 211198; 1. Nanjing Institute for Food and Drug Control，Nanjing 211198，China; 2.江苏省市场监管局重点实验室（食品真实性鉴别技术），江苏 南京 211198; 2. Key Laboratory for Food Authenticity Identification Technology of Jiangsu Provincial Market Regulation Administration，Nanjing 211198，China; 3.东部战区空军医院普外科，江苏 南京 210001; 3. The Air Force Hospital from Eastern Theater Command，Nanjing 210001，China

**Keywords:** 牛乳过敏原, 超高效液相色谱-串联质谱法, 多肽, 非标定量, 低致敏婴幼儿配方乳粉, cow’s milk allergens （CMAs）, ultra-high performance liquid chromatography-tandem mass spectrometry （UHPLC-MS/MS）, peptides， label-free quantification （LFQ）, hypoallergenic infant formula

## Abstract

本研究以低致敏婴幼儿配方乳粉为研究对象，结合抗原表位特征序列和质谱响应，对先前挖掘的18条表征牛乳过敏蛋白的特征多肽进一步筛选，得到6条定量多肽。随后建立了同时定量测定低致敏婴幼儿配方乳粉中6种牛乳致敏蛋白（*α*-乳白蛋白、*β*-乳球蛋白、*α*S1-酪蛋白、*α*S2-酪蛋白、*β*-酪蛋白及*κ*-酪蛋白）的超高效液相色谱-串联质谱（UHPLC-MS/MS）分析方法。添加牛乳致敏蛋白标准品的空白低致敏婴幼儿配方乳粉经过蛋白提取、酶解和净化后进行UHPLC-MS/MS分析。根据每种蛋白不同添加浓度和质谱响应建立标准曲线。结果表明：6种牛乳致敏蛋白在0.05~500 mg/kg内线性范围良好，相关系数（*r* ^2^）均大于0.99，检出限为0.05~5.0 mg/kg，定量限为0.1~10.0 mg/kg，优于文献报道的3种基于质谱和2种基于酶联免疫吸附试验（ELISA）的检测方法。在低、中、高3个添加水平下，6种牛乳致敏蛋白的加标回收率为74.8%~93.4%，日内准确度为-25.09%~-6.09%，日间准确度为-24.0%~-5.41%；日内精密度为1.08%~5.05%，日间精密度为1.35%~4.85%。运用该方法对市售低致敏婴幼儿配方乳粉进行检测，3种部分水解低致敏婴幼儿配方乳粉（pHF）和2种深度水解低致敏婴幼儿配方乳粉（eHF）检出牛乳过敏蛋白，检出量为0.48~27.36 mg/kg。本方法灵敏度好，通量高，可用于低致敏婴幼儿配方乳粉中牛乳过敏原的定量筛查。

## Abstract (en)

Cow’s milk protein allergy （CMPA） has become a common public health concern and hypoallergenic formula （partially or extensively hydrolyzed formula） is considered as an alternative during infancy. Alcalase and papain are widely used in industrial production of hydrolysates， as they can modify or destroy the linear and conformational epitopes of cow’s milk allergens （CMAs）， thus leading to decreased allergenicity. However， despite considerable emphasis has been laid on the manufacturing technique and nutritional value of hypoallergenic formulas， whether there are traces of CMAs in hypoallergenic formulas， remains unknown. Consequently， the need for a quantitative method of detecting multiple CMAs at a time is rising. Traditionally， enzyme-linked immunosorbent assay （ELISA）-based methods have proved to be advantageous with a wide availability of commercial kits for CMAs detection. Moreover， polymerase chain reaction （PCR）， as one of DNA-based methods， has shown exceptional specificity and high sensitivity. However， they both may encounter false-positive results and high-throughput detection is challenging. In the past two decades， mass spectrometry （MS）-based proteomic technology has advanced， including improvements in computational capability， algorithm model， as well as mass spectrometric platforms. MS is gaining more and more attention in the field of food allergen detection， allowing multiplex allergen detection with high sensitivity， accuracy and selectivity in a single run. In this study， an analytical method was developed for the simultaneous quantitation of six CMAss （namely， *α*-lactalbumin， *β*-lactoglobulin， *α*S1-casein， *α*S2-casein， *β*-casein， and *κ*-casein） in hypoallergenic formulas using ultra-high performance liquid chromatography-tandem mass spectrometry （UHPLC-MS/MS）. Proteins of spiked hypoallergenic formula were extracted with ammonium bicarbonate and digested with trypsin. After digestion， the samples were purified with a C_18_ spin column， and analyzed by UHPLC-MS/MS （AB SCIEX Triple Quad 6500^+^）. Separation was achieved on a Waters ACQUITY UPLC Peptide CSH C_18_ column （150 mm×2.1 mm， 1.7 μm）. The mobile phase consisted of 0.1% formic acid aqueous solution and 0.1% formic acid in acetonitrile， and the flow rate was 0.3 mL/min. The sample injection volume was 10 μL， and the column temperature was set to 40 ℃. Mass spectrometer was operated in positive electrospray ionization （ESI^+^） mode， and multiple reaction monitoring （MRM） mode was adopted. Precursor ions and product ions of marker peptides were predicted by the Skyline software. Mass spectrometric parameters such as declustering potential （DP） and collision energy （CE） were also optimized. Afterwards， by taking allergen epitopes and mass spectrometric responses into consideration， six peptides were retained for quantitative analysis. Calibration curves were constructed with good linearity in the range of 0.05-500 mg/kg， and correlation coefficients （*r* ^2^） greater than 0.99. The limits of detection （LODs） were 0.05-5.0 mg/kg， and limits of quantification （LOQs） were 0.1-10.0 mg/kg， respectively. The recoveries of the developed method in incurred hypoallergenic formulas ranged from 74.8% to 93.4%. The intraday and interday accuracies ranged from -25.09% to -6.09% and from -24.0% to -5.41%， with precisions between 1.08%-5.05% and 1.35%-4.85%， respectively. Screening of commercial hypoallergenic formula samples confirmed existence of CMAs in three partially hydrolyzed formulas （pHF） and two extensively hydrolyzed formulas （eHF）， with contents ranging from 0.48 to 27.36 mg/kg. The analytical method proposed in this study exhibited high sensitivity， high-throughput capability， and can be used to monitor traces of CMAs in hypoallergenic formulas.

婴幼儿主要以母乳作为营养来源，当母乳不足或者无法获得时，通常需要在食物中补充以牛乳为主要原料的婴幼儿配方乳粉，这可能会引起牛乳过敏（cow’s milk protein allergy，CMPA）。据报道牛乳过敏婴幼儿约占全球婴幼儿人口的2%~3%，因为婴幼儿身体发育尚未完全，牛乳过敏严重影响其生长发育并给家庭抚养带来负担^［1，2］^。对于牛乳过敏，低致敏配方婴幼儿乳粉通常被认为是合适的替代方案。此外，对于严重过敏或者对低致敏配方乳粉亦不能耐受的婴幼儿，氨基酸配方（amino acid formulas，AAF）粉也被添加到饮食中^［3］^。

牛乳中蛋白含量大约为3 g/100 mL，包括至少25种蛋白，这些蛋白都有可能成为牛乳过敏原（cow’s milk allergens，CMAs）。根据低pH时溶解性不同，牛乳蛋白可以分为两大类：酪蛋白（80%）和乳清蛋白（20%）。酪蛋白在pH=4.6时不可溶，主要由*α*S1-、*α*S2-、*β*-和*κ*-酪蛋白（casein，CN）以4∶1∶4∶1比例构成；而乳清蛋白在pH=4.6时可溶，主要由包括*α*-乳白蛋白（*α*-lactalbumin， ALA）、*β*-乳球蛋白（*β*-lactoglobulin， BLG）等球蛋白组成，还包括少量的牛血清蛋白（bovine serum albumin，BSA）、乳铁蛋白、乳过氧化物酶和免疫球蛋白^［4］^。牛乳蛋白过敏反应可分为免疫球蛋白E（Ig-E）介导的和非Ig-E介导的两大类。过敏反应是由牛乳蛋白中抗原结合表位和T细胞表面受体或者是巨噬细胞表面Ig-E受体结合而触发。抗原表位通常由8~26个氨基酸（AA）组成，分为线性表位和构象表位两类，可分别被T细胞或者B细胞表面的受体识别^［5-7］^。研究表明修饰牛乳蛋白的抗原表位可以降低其致敏性，这就是低致敏婴幼儿配方乳粉生产工艺的基础。自1985年第一款部分/适度水解配方乳粉（Beba HA、Good start和NAN HA，雀巢公司）上市以来，很多处理工艺，包括加热、高压、酶解、超声波和糖基化等，都被用来生产低致敏婴幼儿配方乳粉。其中，酶水解工艺最为常见。这一工艺通过将大分子牛乳蛋白水解，降低了牛乳蛋白的致敏性。根据水解程度的不同，低致敏配方婴幼儿乳粉可以分为部分水解婴幼儿配方乳粉（partially hydrolyzed formulas， pHF）和深度水解婴幼儿配方乳粉（extensively hydrolyzed formulas， eHF）。总的来说，pHF由分子质量为3~10 kDa的多肽组成，而eHF中肽段90%由分子质量小于3 kDa（大多数<1.5 kDa）的短肽和氨基酸组成^［8，9］^。值得注意的是，酶解过程有时候不能完全破坏抗原表位，并且酶解等工艺也可能导致新的抗原表位被暴露出来，增加牛乳蛋白的致敏性，因此对于低致敏婴幼儿配方乳粉中是否残留痕量的牛乳过敏原，亟需建立一种分析方法。

对于牛乳过敏原检测，传统的方法有基于酶联免疫吸附试验（ELISA）检测方法和基于聚合酶链式反应（PCR）方法两大类。ELISA方法操作简单，商业化程度高，但是容易出现假阳性结果且难以实现高通量检测；PCR方法具有高灵敏度和高特异性的特点，但是其对于DNA的检测并不能真实反映致敏蛋白的存在，降低了结果的准确性。近年来，诸如表面等离子体共振（SPR）、生物传感器（biosensor）和等电聚焦微阵列芯片（mIEF）等技术在食品过敏原检测方面得到了一些应用，但是这些方法大规模应用还有待进一步验证^［10-12］^。

自从第一个运用LC-MS检测过敏原的研究^［13］^发表以来，质谱技术被越来越多地应用到食品过敏原检测中^［14-18］^。对于食品中过敏原含量测定，传统方法有运用同位素标记多肽的绝对定量技术（absolute quantification，AQUA），这种定量技术虽然可以对过敏原蛋白进行准确定量，但是成本较高。近年来，非标定量技术（label-free quantification，LFQ）受到了研究人员的青睐，这一技术通过比较同一蛋白产生的MS图谱或者离子的响应来完成定量，这一定量方法相对于AQUA成本低廉，但是批次间重复性和精密度需要验证，此外定量多肽的选择也会影响过敏蛋白含量的测定^［19-23］^。

本研究在先前研究^［24］^定性测定的基础上，结合抗原表位和质谱响应，对18条表征6种牛乳过敏蛋白的特征多肽进行了进一步筛选，得到6条定量特征多肽，随后以添加牛乳蛋白标准品的空白深度水解配方乳粉作为基质，建立了基于超高效液相色谱-串联质谱（UHPLC-MS/MS）同时测定低致敏婴幼儿配方乳粉中6种牛乳致敏蛋白的定量分析方法。本研究建立的分析方法可以为低致敏婴幼儿配方乳粉中痕量牛乳过敏原检测提供方法学依据，同时可以为相关乳粉生产企业以及监管部门提供技术支撑。

## 1 实验部分

### 1.1 仪器、试剂与材料

Triple Quad 6500^+^超高效液相色谱-三重四极杆质谱仪（美国AB SCIEX公司）；Milli-Q IQ7000超纯水系统（美国Millipore公司）；5425R小型冷冻离心机（德国Eppendorf公司）；LYNX4000离心机、TSGP20恒温水浴锅（美国Thermo Fisher公司）；涡旋振荡仪（德国Heidolph公司）；ME503T型电子天平（瑞士Mettler Toledo公司）。

二硫苏糖醇（DL-dithiothreitol， DDT）、碘乙酰胺（iodoacetamide， IAA）、三氟乙酸（trifluoroacetic acid，TFA，色谱级）、考马斯亮蓝G250、牛血清白蛋白（分析级）购自中国阿拉丁公司；碳酸氢铵（ammonium bicarbonate， ABC， 色谱级）、冰乙酸（色谱级）、三羟甲基氨基甲烷-盐酸（Tris-HCl）、乙腈、甲醇、甲酸（均为质谱级）购自美国Honeywell公司；胰蛋白酶购自美国Sigma公司；ALA（94%）、BLG标准品（99%）、*α*-CN（76%）（*α*S1-CN与*α*S2-CN含量比为4∶1）、*β*-CN（100%）、*κ*-CN标准品（92%）均购自美国Sigma公司。聚醚砜（PES）滤膜（0.22 μm）购自爱尔兰Merck Millipore公司。实验使用超纯水（18.2 MΩ·cm）均由Milli-Q超纯水仪制备。实验用低致敏婴幼儿配方乳粉均购于江苏南京当地母婴用品店。

### 1.2 溶液配制和阳性样品制备

#### 1.2.1 牛乳过敏蛋白标准溶液配制

分别准确称取ALA、BLG、*α*-CN、*β*-CN和*κ*-CN标准品10 mg于10 mL棕色容量瓶中，用1%乙酸溶液定容至刻度，得到质量浓度为1.0 mg/mL的储备液，存于-80 ℃备用。使用前将ALA、BLG、*α*-CN和*β*-CN储备液用超纯水稀释至100 μg/mL。

#### 1.2.2 阳性样品制备

选用事先经过提取、酶解并且上机分析后确证不含有牛乳过敏蛋白的某品牌深度水解配方乳粉作为空白基质。准确称取0.2 g空白基质乳粉，加入牛乳过敏蛋白储备液（ALA、BLG、*α*-CN和*β*-CN： 100 μg/mL；*κ*-CN： 1 mg/mL），得到ALA和BLG添加水平为1、2、5、10、20、50、100 mg/kg，*α*S1-CN添加水平为0.2、0.4、1.0、2.0、4.0、10.0、20.0 mg/kg，*α*S2-CN添加水平为0.05、0.1、0.2、0.5、1.0、2.0、5.0 mg/kg，*β*-CN添加水平为0.1、0.2、0.5、1.0、2.0、5.0、10.0 mg/kg，*κ*-CN添加水平为5、10、20、50、100、250、500 mg/kg （mg总蛋白/kg空白基质）。

### 1.3 样品前处理

准确称取0.2 g低致敏配方婴幼儿乳粉于15 mL聚丙烯离心管中，加入10 mL 100 mmol/L ABC，25 ℃超声提取10 min，在15 000 g、4 ℃下离心10 min，离心后小心去除上清脂肪，测定蛋白含量后移取200 μL上清液于2 mL离心管中，加入150 μL 100 mmol/L ABC和10 μL DTT，70 ℃水浴30 min，取出冷却后加入30 μL 100 mmol/L IAA，25 ℃暗处放置30 min，随后加入20 μL 1 μg/μL胰蛋白酶溶液，37 ℃酶解12 h后加入0.5 mL TFA终止反应，随后，将酶解产物在15 000 g、4 ℃下离心5 min后过0.22 μm PES滤膜后置于进样小瓶中，待分析。

### 1.4 仪器分析条件

#### 1.4.1 色谱条件

选用Waters ACQUITY UPLC Peptide CSH C_18_色谱柱（150 mm×2.1 mm， 1.7 μm）对酶解后牛乳过敏蛋白多肽进行分离。流动相A为0.1%甲酸乙腈，流动相B为0.1%甲酸水溶液。进样量为10 μL，流速为0.3 mL/min，柱温为40 ℃。流动相梯度洗脱程序：0~1 min，90%B；1~15 min，90%B~60%B； 15~15.1 min，60%B~0B； 15.1~18 min，0B； 18~18.1 min，0B~90%B； 18.1~20 min，90%B。

#### 1.4.2 质谱条件

离子源：电喷雾离子源；电离模式：正离子模式；离子源温度（TEM）：500 ℃；气帘气压力（CUR）：241.3 kPa；碰撞气压力（CAD）：62.1 kPa；喷雾电压（IS）：5 000 V；采集方式：多反应监测（MRM）模式；MRM参数、去簇电压（DP）和碰撞能量（CE）见表1。

**表1:** 6种CMAs定量特征多肽的质谱参数

Compound	Sequence	Precursor ion （*m/z*）	Product ions （*m/z*）	*t*_R_ /min	DP/eV	CE/eV
ALA	VGINYWLAHK	600.83	817.44， 654.37， 931.48^*^	8.32	60.0	28.4
BLG	VLVLDTDYK	533.30	641.28， 754.36， 853.43^*^	7.33	60.0	25.1
*α*S1-CN	FFVAPFPEVFGK	692.87	823.44， 991.53， 920.49^*^	13.69	60.0	33.0
*α*S2-CN	FALPQYLK	490.28	551.32， 761.46， 648.37^*^	9.46	60.0	23.0
*β*-CN	VLPVPQK	390.75	471.29， 681.43， 568.35^*^	3.94	60.0	18.1
*κ*-CN	SPAQILQWQVLSNTVPAK	990.54	1056.61， 1242.68， 1370.74^*^	11.53	40.0	47.5

DP： declustering potential； CE： collision energy； * quantitative ion.

## 2 结果与讨论

### 2.1 质谱条件优化

利用Skyline软件模拟酶切位点，每个母离子肽段选取相应离子对监测，以响应值最高的离子作为定量离子，其余离子作为定性离子，通过UHPLC-MS/MS在Scheduled MRM模式下，选取合适的保留时间，优化DP和CE。离子对信息见表1。

### 2.2 定量特征多肽筛选

在过敏蛋白特征多肽定量肽段的选取过程中，之前研究往往以特征多肽质谱特性（离子响应强度）作为主要依据，大量研究表明过敏由免疫球蛋白（通常是Ig-E）识别食物过敏原表位并引发免疫反应引起。基于上述观点，本研究进一步对先前研究^［24］^中筛选出的18条表征6种CMAs的多肽进行了筛选。根据已有报道^［25，26］^结合序列比对，ALA的特征肽段VGINYWLAHK（AA118~127），包含在预测线性表位DIMCVKKILDKVGINYWLAHKALCSEK（AA107~133）里，所以选择特征多肽VGINYWLAHK为定量多肽；BLG的特征肽段TPEVDDEALEK（AA141~151）与预测线性表位EQSLACQCLVRTP（AA130~142）少量部分重合，特征肽段VLVLDTDYK（AA108~116）与预测线性表位ENKVLVLDT（AA105~113）绝大部分都重合，所以选择特征多肽VLVLDTDYK为定量多肽；*α*S1-CN的特征肽段YLGYLEQLLR（AA106~115）包含在预测线性表位SERYLGYLEQLLRLKKYKVPQLEIVPNS（AA103~130）中，FFVAPFPEVFGK（AA38~49）与预测线性表位LRFFVAPFPEVF（AA36~47）绝大部分都重合，EDVPSER（AA99~105）与预测线性表位SERYLGYLEQLLRLKKYKVPQLEIVPNS（AA103~130）少量部分重合，EGIHAQQK（AA106~115）包含预测线性表位HAQQ（AA143~146）；虽然特征肽段YLGYLEQLLR完全被包含于线性表位中，EGIHAQQK完全包含于线性表位中，但上机发现离子响应强度不高，所以本实验选取与线性表位绝大部分都重合的FFVAPFPEVFGK特征肽段作为定量肽段；*α*S2-CN的特征肽段NAVPITPTLN（AA130~140）与预测线性表位RNANPITP（AA129~136）部分重合，FALPQYLK（AA189~196）被包含于预测线性表位FLKKISQRYQKFALPQYLKTVYQHQ（AA178~202）中，而TVYQHQK（AA197~203）则与预测线性表位FLKKISQRYQKFALPQYLKTVYQHQ（AA178~202）部分都重合，所以选择特征多肽FALPQYLK为定量多肽。*β*-CN的预测线性表位HQPHQPLPP TVMFPPQSVLSLSQSKVLPVPQKAVPYP（AA160~196）包含特征多肽VLPVPQK（AA185~191），并与特征多肽AVPYPQR（AA192~198）部分重合，FQSEE QQQTEDELQDK（AA48~63）与线性表位DKIHPFA QTQSLVYPFPGPIPNS（AA62~84）极少量部分重合，因此选择特征多肽VLPVPQK为定量肽段。*κ*-CN的特征肽段SPAQILQWQVLSNTVPAK（AA90~107）与预测线性表位PVALINNQFLPYPYYAKPAAVRSPAQ ILQWQV（AA68~99）部分重合，因此选择特征多肽SPAQILQWQVLSNTVPAK为定量肽段。如表1所示，每种CMAs选取1条特征多肽，共计6条。在选择特征多肽并建立食品过敏原定量分析方法时考虑抗原表位增加了方法的特异性。随后上机分析，如图1所示，各条多肽峰形良好，可以满足分析要求。

**图1:**
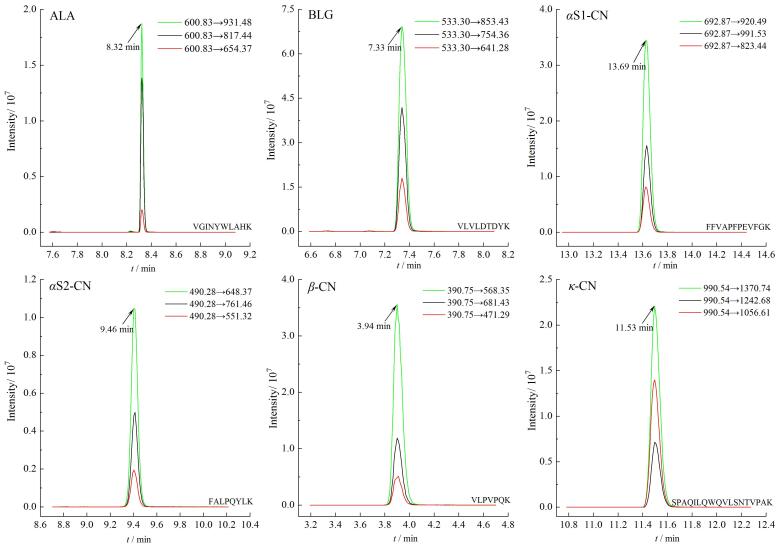
6种CMAs定量特征多肽的MRM色谱图

### 2.3 方法学考察

#### 2.3.1 线性范围、检出限和定量限

在优化的条件下，通过在不含有CMAs的深度水解配方乳粉中添加CMAs模拟阳性样品的方式，将提取酶解后的蛋白上机，以CMAs添加水平为*x*，特征多肽峰面积为*y*，绘制标准曲线。结果显示，6种CMAs在0.05~500 mg/kg范围内线性关系良好，相关系数（*r*^2^）均达到0.99以上。分别以3倍和10倍信噪比（*S/N*）所对应的6种CMAs质量浓度作为检出限（LOD）和定量限（LOQ）。结果如表2所示，6种CMAs的LOD和LOQ分别为0.05~5.0 mg/kg和0.1~10.0 mg/kg。

**表2:** 6种CMAs的线性方程、相关系数、检出限和定量限

Compound	Sequence	Linear equation	*r* ^2^	LOD/（mg/kg）	LOQ/（mg/kg）
ALA	VGINYWLAHK	*y*=4.5181×10^5^ *x*+1.9274×10^5^	0.9989	1.0	2.0
BLG	VLVLDTDYK	*y*=2.3897×10^5^ *x*+2.7569×10^5^	0.9996	1.0	2.0
*α*S1-CN	FFVAPFPEVFGK	*y*=9.5596×10^4^ *x*+1.2213×10^4^	0.9989	0.2	0.4
*α*S2-CN	FALPQYLK	*y*=5.4349×10^4^ *x*-6.7579×10^2^	0.9995	0.05	0.1
*β*-CN	VLPVPQK	*y*=3.8697×10^4^ *x*-1.603×10^3^	0.9994	0.1	0.2
*κ*-CN	SPAQILQWQVLSNTVPAK	*y*=1.8437×10^4^ *x*+7.6226×10^4^	0.9996	5.0	10.0

*y*： peak area； *x*： content of CMAs spiked into blank matrices， mg/kg.

将本研究中获得的LOD和LOQ与文献中报道的值进行比较，如表3所示，本方法的LOD和LOQ值折算质量浓度（以*α*S2-CN作为基础折算）分别为1.0 ng/mL和2.0 ng/mL，低于2种基于质谱和3种基于ELISA的检测方法^［27-32］^。并且，相对于文献报道的同时测定一种或几种CMAs的方法，本方法能一次性覆盖6种CMAs，可以对无论是添加全牛乳或是部分添加牛乳蛋白（例如乳清蛋白）的样品进行测定。

**表3:** 本方法与已报道方法的比较

Method	LFQ	Matrices	Analytes	Linear range	LOD	LOQ	Ref.
LC-MS/MS	Y	hypoallergenic formula	ALA， BLG， *α*S1-CN， *α*S2-CN， *β*-CN， *κ*-CN	0.05‒50 mg/kg	0.05 mg/kg （equiv. to 1.0 ng/mL）	0.1 mg/kg （equiv. to 2.0 ng/mL）	this work
LC-MS/MS	Y	hypoallergenic formula	*α*S1-CN， *α*S2-CN	1.8‒42 mg/kg	0.4 mg/kg	1.3 mg/kg	［^27^］
LC-MS/MS	N	baked food	*α*S1-CN， *α*S2-CN， *β*-CN， *κ*-CN	1‒100 nmol/L	0.12 mg/kg	0.38 mg/kg	［^28^］
LC-MS/MS	N	cake， cookie	ALA， BLG， *α*S1-CN	0.97‒31.25 μg/mL	0.20 μg/mL	0.48 μg/mL	［^29^］
ELISA	/	hypoallergenic formula	ALA	61.04 ng/mL‒ 62.50 μg/mL	1.59 ng/mL	1.61 ng/mL	［^30^］
ELISA	/	yoghurt， chocolate， milk， candy	BLG	31.25‒8000 ng/mL	1.96 ng/mL	3.91 ng/mL	［^31^］
Biosensor	/	infant food formula	BLG	0.01‒1000 ng/mL	0.007 ng/mL	/	［^32^］

LFQ： label-free quantification； Y： yes； N： no. LODs and LOQs were compared using the lowest CMAs category level achieved in respective studies.

#### 2.3.2 回收率和精密度

分别在空白深度水解配方乳粉中进行低、中、高3个水平的CMAs加标回收试验，按照1.3节方法进行蛋白提取酶解后上机分析，考察方法的回收率。如表4所示，6种CMAs在3个添加水平下回收率范围为74.8%~93.4%，RSD为2.3%~4.9%。

**表4:** 不同添加水平下6种CMAs的加标回收率和相对标准偏差（*n*=6）

Compound	Spiked level/（mg/kg）	Recovery/%	RSD/%
ALA	1 5 10	81.2 85.6 91.9	2.9 3.0 3.4
BLG	1 5 10	82.9 87.2 93.4	4.8 3.8 3.5
*α*S1-CN	0.2 1.0 2.0	80.1 86.9 92.5	4.8 4.3 3.9
*α*S2-CN	0.05 0.25 0.5	79.7 83.3 90.9	4.7 3.3 2.8
*β*-CN	0.1 0.5 1.0	81.4 84.7 89.1	4.9 3.9 2.3
*κ*-CN	5.0 25.0 50.0	74.8 80.4 85.5	3.0 3.3 3.1

此外，在空白深度低致敏配方婴幼儿乳粉基质中添加低、中、高3个水平（ALA和BLG为1.0、2.0、10.0 mg/kg；*α*S1-CN为0.2、0.4、2.0 mg/kg；*α*S2-CN为0.05、0.1、0.5 mg/kg；*β*-CN为0.1、0.2、1.0 mg/kg；*κ*-CN为5.0、10.0、50.0 mg/kg）的6种CMAs标准品，按照1.3节方法进行蛋白提取酶解后上机分析，每个水平设置6组平行样本。于同一天内对样本进行连续进样，并且连续3天对样本进行进样测定，以相对误差（relative error， RE）表征准确度，以RSD表征精密度，考察方法的日内及日间的准确度及精密度。结果表明，6种CMAs在3个添加水平下日内准确度为-25.1%~-6.09%，精密度为1.08%~5.05%；日间准确度为-24.0%~-5.41%，精密度为1.35%~4.85%。综上所述，实验建立的方法具有较好的准确度和精密度，可以满足低致敏配方乳粉中6种CMAs痕量分析的要求。

### 2.4 实际样品分析

运用本研究建立的分析方法对市售10批次低致敏婴幼儿配方乳粉（5批次为部分水解配方乳粉，5批次为深度水解配方乳粉）进行检测，如表5所示，编号为1、3和5号的pHF样品中检出CMAs蛋白，提示这3个批次乳粉中存在尚未水解完全的牛乳蛋白，这一结果是因为和含有完整牛乳蛋白的配方乳粉相比，pHF通过对牛乳蛋白进行部分水解而降低其致敏性，旨在提高牛乳过敏婴幼儿的耐受性。另一方面，在编号为6和8的eHF样品中检测到乳清蛋白，提示可能存在CMAs，这可能来源于生产过程中无意带入，这些微量的牛乳蛋白也可能引起婴幼儿致敏反应，基于eHF相对于pHF应该水解更加彻底，这些检出的微量CMAs也应该引起重视。这些结果表明本方法可以运用于实际低致敏婴幼儿配方乳粉样品中CMAs的筛查，并且本方法可以确证样品中含有的CMAs种类，因为在有些情况下，生产过程中部分添加了乳清蛋白或者酪蛋白，而不是全牛乳。

**表5:** 实际样品中6种CMAs的含量 (mg/kg)

Compound	Partially hydrolyzed formulas （pHF）					Extensively hydrolyzed formulas （eHF）				
	No. 1	No. 2	No. 3	No. 4	No.5	No. 6	No. 7	No. 8	No. 9	No. 10
ALA	9.86	ND	5.23	ND	10.30	23.74	ND	5.23	ND	ND
BLG	7.34	ND	3.80	ND	6.82	16.72	ND	2.78	ND	ND
*α*S1-CN	1.74	ND	1.27	ND	3.54	ND	ND	ND	ND	ND
*α*S2-CN	0.62	ND	0.48	ND	1.82	ND	ND	ND	ND	ND
*β*-CN	2.47	ND	1.58	ND	2.76	ND	ND	ND	ND	ND
*κ*-CN	27.36	ND	15.26	ND	22.17	ND	ND	ND	ND	ND
ALA	9.86	ND	5.23	ND	10.30	23.74	ND	5.23	ND	ND

ND： not detected.

## 3 结论

低致敏婴幼儿配方乳粉相对于普通配方婴幼儿乳粉，价格昂贵，是CMAs过敏婴幼儿的主要乳粉品种，对于处于生长发育初期的婴幼儿来说，其中是否含有痕量牛乳过敏原至关重要。先前大量研究集中在如何通过酶解、加热或者超声等手段降低牛乳蛋白致敏性。而对于这一特殊基质中是否含有痕量牛乳过敏原，缺乏筛查和定量的分析方法。本研究将过敏原表位和质谱响应相结合，从之前研究报道的表征牛乳过敏蛋白的18条特征多肽中筛选出6条多肽，随后通过在空白深度水解低致敏婴幼儿配方乳粉中添加牛乳蛋白标准品的方式，建立了低致敏婴幼儿配方乳粉中6种CMAs的UHPLC-MS/MS定量分析方法。该方法具有较低的检出限，良好的准确度和精密度，可一次性测定6种CMAs，满足低致敏婴幼儿配方乳粉中痕量CMAs检测的分析要求。实际样品分析表明在深度水解婴幼儿配方乳粉中可能存在牛乳致敏蛋白残留，这可能给牛乳过敏婴幼儿带来致敏风险，当然本研究样本量有限，还需要进一步对市场中所售低致敏婴幼儿配方乳粉进行筛查，掌握更多数据，了解牛乳致敏蛋白残留情况。
